# Morphological characterizations of parsnip (*Pastinaca sativa* L.) to select superior genotypes

**DOI:** 10.1002/fsn3.3371

**Published:** 2023-04-19

**Authors:** Ali Khadivi, Farhad Mirheidari, Younes Moradi

**Affiliations:** ^1^ Department of Horticultural Sciences, Faculty of Agriculture and Natural Resources Arak University Arak Iran

**Keywords:** conservation, cultivation, parsnip, *Pastinaca sativa* L., quality, superior

## Abstract

Parsnip (*Pastinaca sativa* L.) is an edible root that has long been used in cooking and preparing baby food and livestock. The present study was performed to evaluate the phenotypic diversity of 69 accessions of this species to select superiors in terms of root quality in Paykan village, Isfahan province, Iran, in the year 2022. There were significant differences among the accessions investigated (ANOVA, *p* < .01). Coefficient of variation (CV) was more than 20.00% in the majority of measured characters (64 out of 66 characters), indicating high diversity among the accessions. Foliage width (crown) ranged from 10 to 55 cm with an average of 32.32 cm. Root shape was tapering (33), obtriangular (10), narrow oblong (5), wide oblong (5), obovate (13), and fusiform (3). Root length ranged from 81.2 to 294 mm with an average of 166.44 mm. Root diameter at its middle point ranged from 15.58 to 125.12 mm with an average of 51.83 mm. Root weight ranged from 15 to 1200 g with an average of 315.36 g. Inner core (xylem) pigmentation/color was cream yellow (11 accessions), light yellow (12), yellow (42), dark yellow (2), and yellow–light orange (2). In the cluster analysis based on Ward's method, the accessions were divided into two main clusters according to morphological traits. This is despite the fact that parsnip is part of the medicinal plant native and valuable in most farms in tropical cities. Compared with carrots, parsnip plants are more adaptable to different environmental conditions. The accessions studied here showed high phenotypic diversity. Based on ideal values of the important and commercial characters of parsnip, such as root length, root weight, inner core (xylem) pigmentation/color, root shape, flesh color intensity, flesh palatability, and total soluble solids, 14 genotypes, including Parsnip‐3, Parsnip‐9, Parsnip‐24, Parsnip‐32, Parsnip‐32, Parsnip‐48, Parsnip‐51, Parsnip‐52, Parsnip‐58, Parsnip‐60, Parsnip‐62, Parsnip‐65, Parsnip‐67, and Parsnip‐69, were promising and are recommended for cultivation.

## Graphical abstract

Parsnip (*Pastinaca sativa* L.) is an edible root that has long been used in cooking and preparing baby food and livestock. The present study was performed to evaluate the phenotypic diversity of 69 accessions of this species to select the superior one in terms of root quality. Based on ideal values of the important and commercial characters of parsnip, such as root length, root weight, inner core (xylem) pigmentation/color, root shape, flesh color intensity, flesh palatability, and total soluble solids, 14 genotypes, including Parsnip‐3, Parsnip‐9, Parsnip‐24, Parsnip‐32, Parsnip‐32, Parsnip‐48, Parsnip‐51, Parsnip‐52, Parsnip‐58, Parsnip‐60, Parsnip‐62, Parsnip‐65, Parsnip‐67, and Parsnip‐69, were promising and are recommended for cultivation.

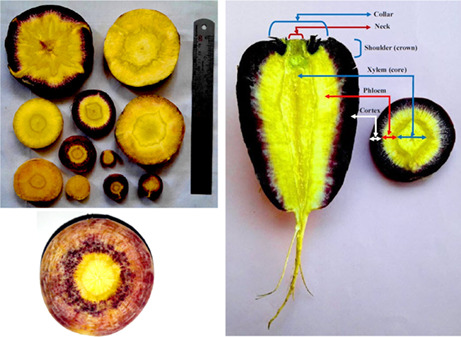

## 1 INTRODUCTION

Parsnip (*Pastinaca sativa* L.) is an edible root that has long been used in cooking and preparing baby food and livestock (Castro et al., 2012). It can also have therapeutic applications depending on the dosage and method of cooking (Stannard, 1982). Parsnip root is high in dietary fiber about 4.70–4.90% (Stannard, 1982). Being rich in starch and sugar, the parsnip root is used for human consumption (in soups, cakes, muffins, and puddings), animal feed, and winemaking. Its fresh leaves and buds are also used as vegetables for food and soups. It has various nutritional and therapeutic applications in different countries. For instance, parsnip is used as an appetizer, digestive, and diuretic in some countries. The seeds of parsnip contain bitter aromatic substances that increase milk in lactating mothers and are also used as food spices; it tastes like dill (Matejić et al., 2014).

*Pastinaca sativa* has different conventional names in different languages such as Zardak and wild carrot in Persian, parsnip in English, Cujtive and Panipainais in French, Jazar in Arabic, and Kajer in India (Emami et al., 2010). From botanical point of view, there is much controversy about distinguishing the parsnip from carrot. Some historians believe the color and taste of the carrots are gradually changed over time; the wild carrots were pale white or yellow and the native carrots were pale yellow or purple. Pale white and yellow carrots may come from mutations in the colored carrot gene. In the 18th century, Linnaeus for the first time provided separate scientific names for them, naming the carrot *Daucus carota* L. and parsnip *Pastinaca sativa*. Galen was the first who explicitly separated carrot from parsnip in his writings (Bahrami et al., 2018; Grant, 2000; Stolarczyk & Janick, 2011).

Wild parsnip is an herbaceous biennial (or more correctly, a monocarpic perennial) within the Apiaceae (Umbelliferae) (carrot, parsley) (Zomlefer, 1994), subfamily Apioideae, tribe Peucedaneae, and subtribe Ferulinae (Peucedaninae), based on Drude (1897–1898) classification of the Apiaceae (Downie et al., 1998). However, Theobald (1971) noted that Pastinaca shares most floral and fruit anatomical characteristics with members of the genus Heracleum, which are classified in the Tordyliinae subtribe. This taxonomic similarity suggests distinct differences within the subtribes of the Peucedaneae.

Wild parsnip is a tall, stout, herbaceous plant with a long, thick, and deep taproot (Gleason & Cronquist, 1991). Wild parsnip is widely distributed in Europe and temperate Asia, where it originated. Wild parsnip is commonly found in waste areas, old fields, and along roadsides and railroad embankments. It grows best in rich, alkaline, and moist soils, but can survive under poor soil conditions (Gleason & Cronquist, 1991). Under summer drought conditions in Oxfordshire, UK, Sternberg et al. (1999) found that the growth of wild parsnip plants in an old field increased and the growth of perennial grasses decreased. This tolerance to drought by wild parsnip may be due to its deep tap root, which allows access to water and nutrients from deeper soil layers (Tutin, 1980).

Wild parsnip plants contain at least seven classes of secondary compounds, including terpenes, flavonoids, polyacetylenes, coumarins, and furanocoumarins (Berenbaum, 1985). Some of the compounds are phenylpropanoids, such as myristicin, which, when combined with xanthotoxin, is synergistically toxic to some insects (Berenbaum, 1985); monoterpenes, which are attractants for pollinators and antimicrobial agents (Harrewijn et al., 1994); sesquiterpenes, which are known to be toxic and deterrents to insects; and fatty acid esters, which are toxic to the larvae of some lepidopterans (Zangerl & Berenbaum, 1993).

Morphological traits are important parameters for the identification and selection of favorable genotypes as plant breeders can use this information for the development of breeding populations (Greene et al., 2004). Both qualitative and quantitative morphological characteristics are useful for germplasm studies. Generally, qualitative parameters are useful for varietal identification, while quantitative parameters are required for the development of new varieties (Luitel et al., 2018). There are no reports about the morphological traits of parsnip. Therefore, a detailed analysis of morphological variability in parsnip core collection is required to understand the diversity in both the qualitative and quantitative parameters. This characterization of morphological parameters is considered an important step in the description and classification of germplasm. Thus, the main objective of the present study was to evaluate morphological characteristics of *P. sativa* in Isfahan province, Iran, to select superiors.

## 2 MATERIALS AND METHODS

### 2.1 Plant material

The present study was performed to evaluate the phenotypic diversity of 69 accessions of parsnip (*P. sativa*) to select superiors in terms of root quality. The accessions studied were cultivated in Paykan village in Isfahan province, Iran, in the year 2022, under homogeneous conditions in loamy clay soil. Paykan village is located at 32°15′50″ N latitude and 52°10′40″ E longitude. The soil of the researched field was analyzed to determine the amount of elements and check the non‐uniformity of the soil. The present experiment was conducted based on a randomized complete block design.

### 2.2 The characteristics evaluated

The phenotypic diversity of the accessions was investigated using 66 morphological traits (Table 1). The traits related to dimensions of leaf and root were measured using a digital caliper. A digital scale with an accuracy of 0.01 g was used to measure the weight of root. Total soluble solids (TSS) were determined using a refractometer (pocket PAL‐1 ATAGO Corporation, Tokyo, Japan), in Brix. The qualitative traits (Table 2) were visually examined and coded according to descriptor of the wild and cultivated carrots (*Daucus carota*) (IPGRI, 1998).

**TABLE 1:** Statistical descriptive parameters for morphological traits used to study *P. sativa* accessions.

No.	Character	Abbreviation	Unit	Min.	Max.	Mean	SD	CV (%)
1	Foliage coverage	V2	Code	1	5	3.52	1.63	46.36
2	Foliage width (crown)	V3	cm	10.00	55.00	32.32	9.15	28.31
3	Leaf growth habit (attitude)	V4	Code	1	5	1.55	0.96	62.13
4	Number of mature leaves per plant	V5	Number	5	47	18.42	8.13	44.13
5	Mature leaf length	V6	mm	110.00	356.00	236.04	51.47	21.81
6	Mature leaf width	V7	mm	40.15	194.34	102.50	31.24	30.48
7	Leaf hairiness	V8	Code	1	7	4.45	1.41	31.66
8	Leaf type	V9	Code	1	7	3.20	1.15	35.78
9	Leaf dissection	V10	Code	1	5	2.07	1.26	61.06
10	Leaflet apex shape	V11	Code	1	5	3.84	1.21	31.46
11	Leaf color	V12	Code	1	9	6.48	2.49	38.41
12	Leaf color intensity	V13	Code	1	5	3.43	1.41	41.08
13	Number of leaflets on lower mature leaf	V14	Number	7	17	10.83	2.63	24.28
14	Length of basal primary leaflet	V15	mm	6.24	141.68	76.09	28.61	37.59
15	Number of segment tips on lower primary leaflet	V16	Number	3	15	6.94	2.50	35.97
16	Petiole length	V17	mm	29.00	201.00	88.48	38.65	43.68
17	Petiole width	V18	mm	2.35	4.90	3.42	0.62	18.24
18	Petiole thickness	V19	mm	1.06	4.45	2.71	0.62	22.67
19	Petiole shape in transverse section	V20	Code	1	5	2.42	1.14	47.23
20	Anthocyanin coloration in petiole	V21	Code	0	5	1.59	1.49	93.58
21	Petiole hairiness	V22	Code	1	7	4.62	1.34	29.03
22	Stem development in first year	V23	Code	1	3	1.03	0.24	23.40
23	Root axis	V24	Code	1	3	2.77	0.65	23.29
24	Root branching	V25	Code	0	3	0.25	0.74	294.40
25	Lateral (secondary) root growth in accession	V26	Code	0	5	0.30	0.83	276.00
26	Emergence of lateral (secondary) roots on fleshy root	V27	Code	0	5	0.51	1.16	227.06
27	Hairy roots on storage root	V28	Code	1	5	1.38	0.99	71.52
28	Emergence of hairy roots on fleshy root	V29	Code	1	5	4.13	1.11	26.88
29	Root length	V30	mm	81.20	294.00	166.44	48.47	29.12
30	Root diameter at the middle point of the root	V31	mm	15.58	125.12	51.83	22.73	43.85
31	Root maximum transverse diameter	V32	mm	20.43	127.33	61.28	23.00	37.53
32	Root maximum transverse diameter position	V33	Code	1	5	1.72	1.49	86.86
33	Root ratio length/diameter	V34	Ratio	1.24	8.34	2.96	1.10	37.09
34	Taproot length	V35	mm	8.73	130.11	49.61	25.80	52.01
35	Neck diameter	V36	mm	8.01	48.77	20.22	7.70	38.09
36	Collar diameter	V37	mm	11.72	76.19	33.97	13.97	41.13
37	Root diameter at shoulder	V38	mm	17.39	103.87	53.89	19.26	35.74
38	Root shoulder shape	V39	Code	1	9	3.93	2.07	52.77
39	Extent of green color of skin on shoulder	V40	Code	0	5	0.68	1.16	170.15
40	Extent of purple color of skin on shoulder	V41	Code	0	5	2.70	2.06	76.30
41	Root weight	V42	g	15	1200	315.36	297.87	94.45
42	Root surface	V43	Code	1	7	2.97	2.18	73.50
43	Root splitting/cracking tendency	V44	Code	0	5	0.23	0.93	402.61
44	Root shape	V45	Code	1	11	3.96	3.49	88.16
45	Root tapering	V46	Code	0	5	3.23	1.81	55.98
46	Root tip/end shape	V47	Code	1	5	4.33	1.07	24.62
47	Root skin pigmentation/color	V48	Code	1	21	12.16	7.30	60.07
48	Root skin color intensity	V49	Code	1	3	2.07	1.01	48.55
49	Inner core (xylem) diameter at shoulder	V50	mm	6.02	64.57	19.74	11.51	58.29
50	Inner core (xylem) diameter at root maximum transverse diameter	V51	mm	6.70	71.88	21.64	13.34	61.67
51	Root diameter of core (xylem) relative to total diameter	V52	Code	1	5	1.55	1.13	73.03
52	Outer core (phloem) thickness at shoulder	V53	mm	3.32	20.32	11.15	4.45	39.90
53	Outer core (phloem) thickness at root maximum transverse diameter	V54	mm	2.88	23.32	12.65	4.97	39.29
54	Outer core (cortex) thickness at root maximum transverse diameter	V55	mm	2.70	14.50	7.54	2.46	32.64
55	Inner core (xylem) pigmentation/color	V56	Code	1	9	4.19	1.79	42.79
56	Outer core (phloem) pigmentation/color	V57	Code	1	21	6.94	4.24	61.12
57	Outer core (cortex) pigmentation/color	V58	Code	1	17	11.64	5.63	48.38
58	Green coloration of interior of the top (xylem)	V59	Code	0	3	0.83	0.82	99.04
59	Green color of outer core (phloem+cortex) at shoulder	V60	Code	0	3	0.64	1.01	158.44
60	White color in outer core (phloem+cortex)	V61	Code	0	1	0.87	0.34	38.97
61	Homogeneity of core pigmentation/coloring throughout root length	V62	Code	1	5	4.59	1.01	21.90
62	Flesh color distribution in transverse section	V63	Code	1	5	2.54	1.20	47.05
63	Homogeneity of flesh coloring throughout root length	V64	Code	1	5	3.72	1.57	42.23
64	Flesh color intensity	V65	Code	1	5	4.30	1.45	33.67
65	Flesh palatability	V66	Code	1	5	3.84	1.47	38.33
66	Total soluble solids	V67	%	7.30	16.50	10.04	1.71	17.02

**TABLE 2:** Frequency distribution for the measured qualitative morphological characteristics in the studied *P. sativa* accessions.

Character	0	1	3	5	7	9	11	13	15	17	19	21
Foliage coverage	‐	16	19	34	‐	‐	‐	‐				
Leaf growth habit (attitude)	‐	Prostrate (51)	Semi‐erect (17)	Erect (1)	‐	‐	‐	‐				
Leaf hairiness	‐	Sparse (2)	Intermediate (23)	Dense (36)	Very dense (8)	‐	‐	‐	‐			
Leaf type	‐	5	55	6	3	‐	‐	‐	‐			
Leaf dissection	‐	Slightly dissected (37)	Intermediate (27)	Highly dissected (5)	‐	‐	‐	‐				
Leaflet apex shape	‐	Round (4)	Semi‐acute (32)	Acute (33)	‐	‐	‐	‐				
Leaf color	‐	Yellow–green (5)	Green (6)	Gray‐green (16)	Blue–green (17)	Purple–green (25)	‐	‐	‐			
Leaf color intensity	‐	Light (11)	Intermediate (32)	Dark (26)	‐	‐	‐	‐				
Petiole shape in transverse section	‐	Round (24)	Semi‐round (41)	Flat (4)	‐	‐	‐	‐				
Anthocyanin coloration in petiole	None (15)	Slightly colored (32)	Intermediate (16)	Strongly colored (6)	‐	‐	‐	‐				
Petiote hairiness	‐	Sparse (1)	Intermediate (20)	Dense (39)	Very dense (9)	‐	‐	‐	‐			
Stem development in first year	‐	Stem consists of a small plate‐like crown (68)	Stem elongates and forms branches (1)	‐	‐	‐	‐					
Root axis	‐	Not straight (8)	Straight (61)	‐	‐	‐	‐	‐				
Root branching	None (60)	Sparse (5)	Intermediate (4)	‐	‐	‐	‐	‐				
Lateral (secondary) root growth in accession	None (56)	Low (10)	Medium (2)	High (1)	‐	‐	‐	‐				
Emergence of lateral (secondary) roots on fleshy root	None (56)	Mostly on upper portion (3)	Mostly on lower portion (9)	All over (1)	‐	‐	‐	‐				
Hairy roots on storage root	‐	Low (59)	Medium (7)	High (3)	‐	‐	‐	‐				
Emergence of hairy roots on fleshy root	‐	Mostly on upper portion (2)	Mostly on lower portion (26)	All over (41)	‐	‐	‐	‐				
Root maximum transverse diameter position	‐	Upper portion (55)	Middle (3)	All over (11)	‐	‐	‐	‐				
Root shoulder shape	‐	Flat (13)	Flat to rounded (23)	Rounded (24)	Rounded to conical (6)	Conical (3)	‐	‐	‐			
Extent of green color of skin on shoulder	None (42)	Low (19)	Intermediate (6)	High (2)	‐	‐	‐	‐				
Extent of purple color of skin on shoulder	None (15)	Low (14)	Intermediate (14)	High (26)	‐	‐	‐	‐				
Root surface	‐	Smooth (30)	Dimpled (21)	Ridged (7)	Raised at the exit of the roots (11)	‐	‐	‐	‐			
Root splitting/cracking tendency	None (63)	Low (3)	Intermediate (1)	High (2)	‐	‐	‐	‐				
Root shape	‐	Tapering (33)	Obtriangular (10)	Narrow oblong (5)	Wide oblong (5)	Obovate (13)	Fusiform (3)	‐	‐	‐		
Root tapering	None (6)	Slight (13)	Intermediate (20)	Acute (30)	‐	‐	‐	‐				
Root tip/end shape	‐	Blunt (2)	Rounded (19)	Pointed (48)	‐	‐	‐	‐				
Root skin pigmentation/color	‐	Yellow–cream (8)	Light yellow (6)	Yellow–light orange (8)	Cream–purple (2)	Yellow–purple (4)	Light orange–purple (1)	Purple–cream (6)	Purple–yellow (8)	Purple–light orange (3)	Purple (10)	Dark purple (13)
Root skin color intensity	‐	Light (32)	Dark (37)	‐	‐	‐	‐	‐				
Root diameter of core (xylem) relative to total diameter	‐	Small (54)	Intermediate (11)	Large (4)	‐	‐	‐	‐				
Inner core (xylem) pigmentation/color	‐	Cream–yellow (11)	Light yellow (12)	Yellow (42)	Dark yellow (2)	Yellow–light orange (2)	‐	‐	‐			
Outer core (phloem) pigmentation/color	‐	Cream (2)	Cream–yellow (16)	Light yellow (13)	Yellow (23)	Dark yellow (4)	Cream–light orange (1)	Yellow–light orange (4)	Cream–purple (2)	Yellow–purple (2)	Purple–yellow (1)	Dark purple (1)
Outer core (cortex) pigmentation/color	‐	Cream–yellow (7)	Yellow–cream (1)	Yellow (10)	Yellow–light orange (3)	Purple–cream (2)	Purple–yellow (2)	Light purple (10)	Purple (13)	Dark purple (21)	‐	
Green coloration of interior of the top (xylem)	None (24)	Weak (39)	Intermediate (6)	‐	‐	‐	‐	‐				
Green color of outer core (phloem+cortex) at shoulder	None (43)	Weak (17)	Intermediate (9)	‐	‐	‐	‐	‐				
White color in outer core (phloem+cortex)	Absent (9)	Present (60)	‐	‐	‐	‐	‐					
Homogeneity of core pigmentation/coloring throughout root length	‐	Low (3)	Intermediate (8)	High (58)	‐	‐	‐	‐				
Flesh color distribution in transverse section	‐	Color in two distinct outer and inner cores (22)	Color radially distributed in stellate pattern (41)	Color radially distributed from inner core (61)	‐	‐	‐	‐				
Homogeneity of flesh coloring throughout root length	‐	Low (13)	Intermediate (8)	High (38)	‐	‐	‐	‐				
Flesh color intensity	‐	Pale/dull (10)	Intermediate (4)	Bright/intense (55)	‐	‐	‐	‐				
Flesh palatability	‐	Low (10)	Intermediate (20)	High (39)	‐	‐	‐	‐				

### 2.3 Statistical analysis

Analysis of variance (ANOVA) was performed to evaluate the variation among accessions based on the traits measured using SAS software (SAS® Procedures., 1990). Simple correlations between traits were determined using Pearson correlation coefficients (SPSS Inc; Norusis, 1998). Principal component analysis (PCA) was used to investigate the relationship between accessions and determine the main traits effective in genotype segregation using SPSS software. The PCA is the simplest of the true eigenvector‐based multivariate analyses. Often, its operation can be thought of as revealing the internal structure of the data in a way that best explains the variance in the data (Iezzoni & Pritts, 1991). Hierarchical cluster analysis (HCA) was performed using Ward's method and Euclidean coefficient with PAST software (Hammer et al., 2001). The first and second principal components (PC1/PC2) were used to create a scatter plot with PAST software.

## 3 RESULTS AND DISCUSSION

There were significant differences among the accessions investigated (ANOVA, *p* < .01). Coefficient of variation (CV) was more than 20.00% in the majority of measured characters (64 out of 66 characters), indicating high diversity among the accessions. The range of CV was from 17.02 (in total soluble solids) to 402.61% (in root splitting/cracking tendency) with an average of 64.69 (Table 1).

**FIGURE 1:**
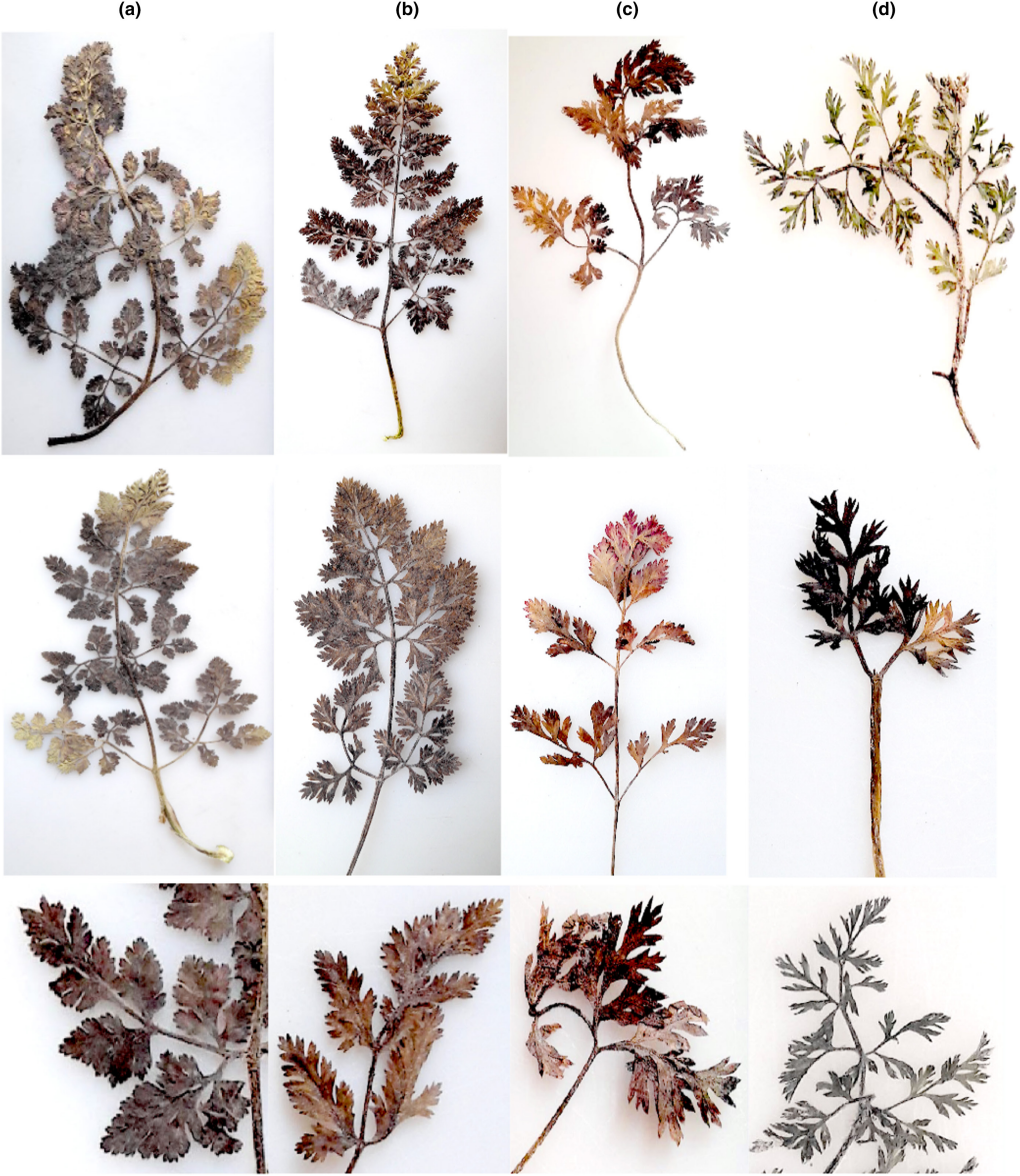
Diversity between *P. sativa* accessions studied in terms of leaf characteristics, including shape; (a) celery, (b) celery but fern form, (c) parsley, (d) carrot.

Foliage width (crown) ranged from 10 to 55 cm with an average of 32.32 (Table 1). Number of mature leaves per plant ranged from 5 to 47 with an average of 18.42. Leaf growth habit (attitude) was prostrate (51 accessions), semi‐erect (17), and erect (1) (Table 2; Figure 1). Mature leaf length varied between 110 and 356 mm. Mature leaf width ranged from 40.15 to 194.34 mm. The average value of petiole length, width, and thickness was 88.48, 3.42, and 2.71 mm, respectively.

**FIGURE 2:**
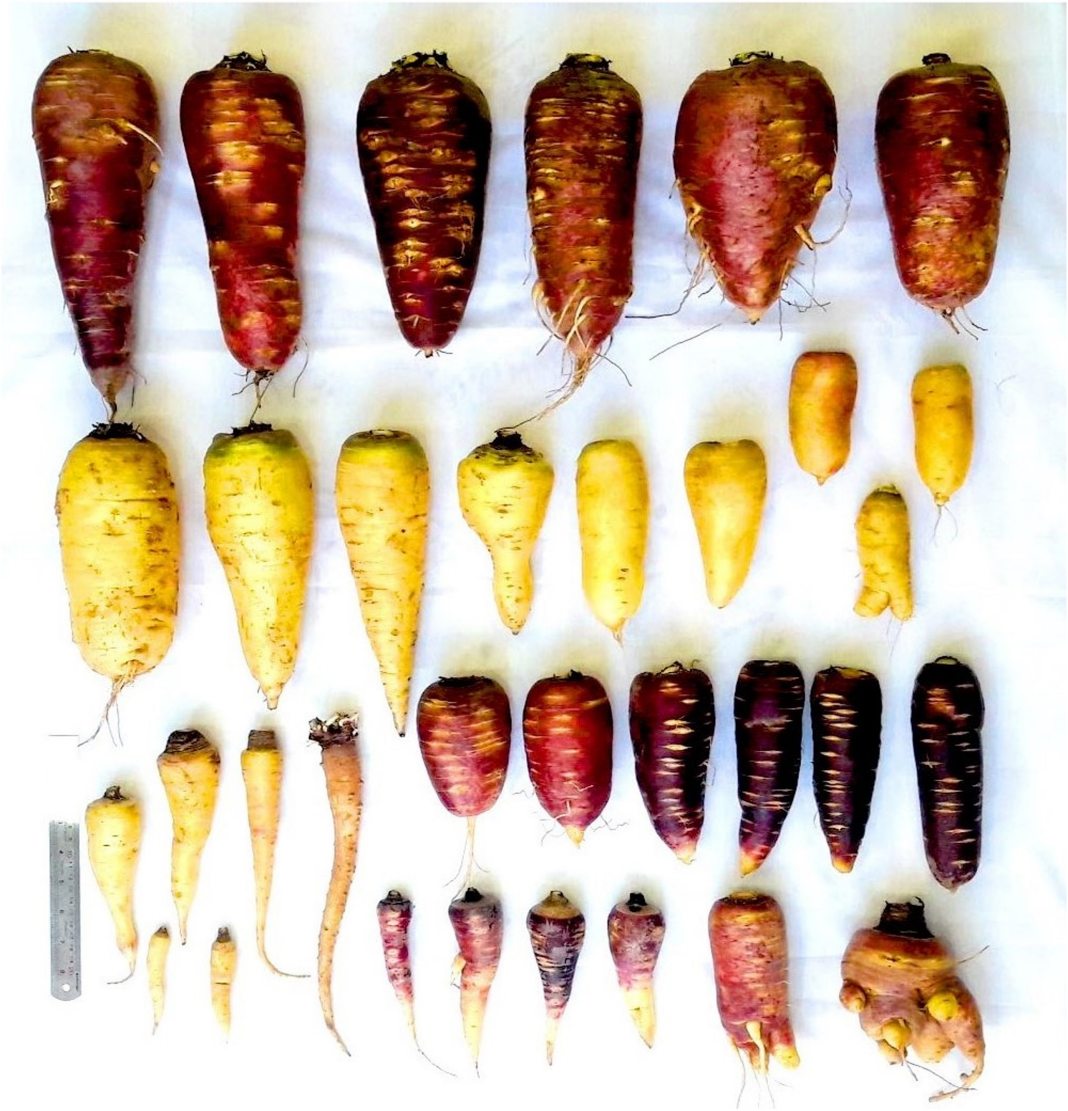
Diversity between *P. sativa* accessions studied in terms of root length, width, and shape.

**FIGURE 3:**
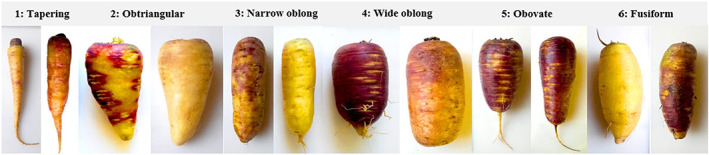
Diversity between *P. sativa* accessions studied in terms of root shape.

Root shape was tapering (33), obtriangular (10), narrow oblong (5), wide oblong (5), obovate (13), and fusiform (3) (Figures 2 and 3). Root length ranged from 81.2 to 294 mm with an average of 166.44 mm. Root diameter at its middle point ranged from 15.58 to 125.12 mm with an average of 51.83 mm. Taproot length varied from 8.73 to 130.11 mm with an average of 49.61 mm. Root weight ranged from 15 to 1200 g with an average of 315.36 g.

**FIGURE 4:**
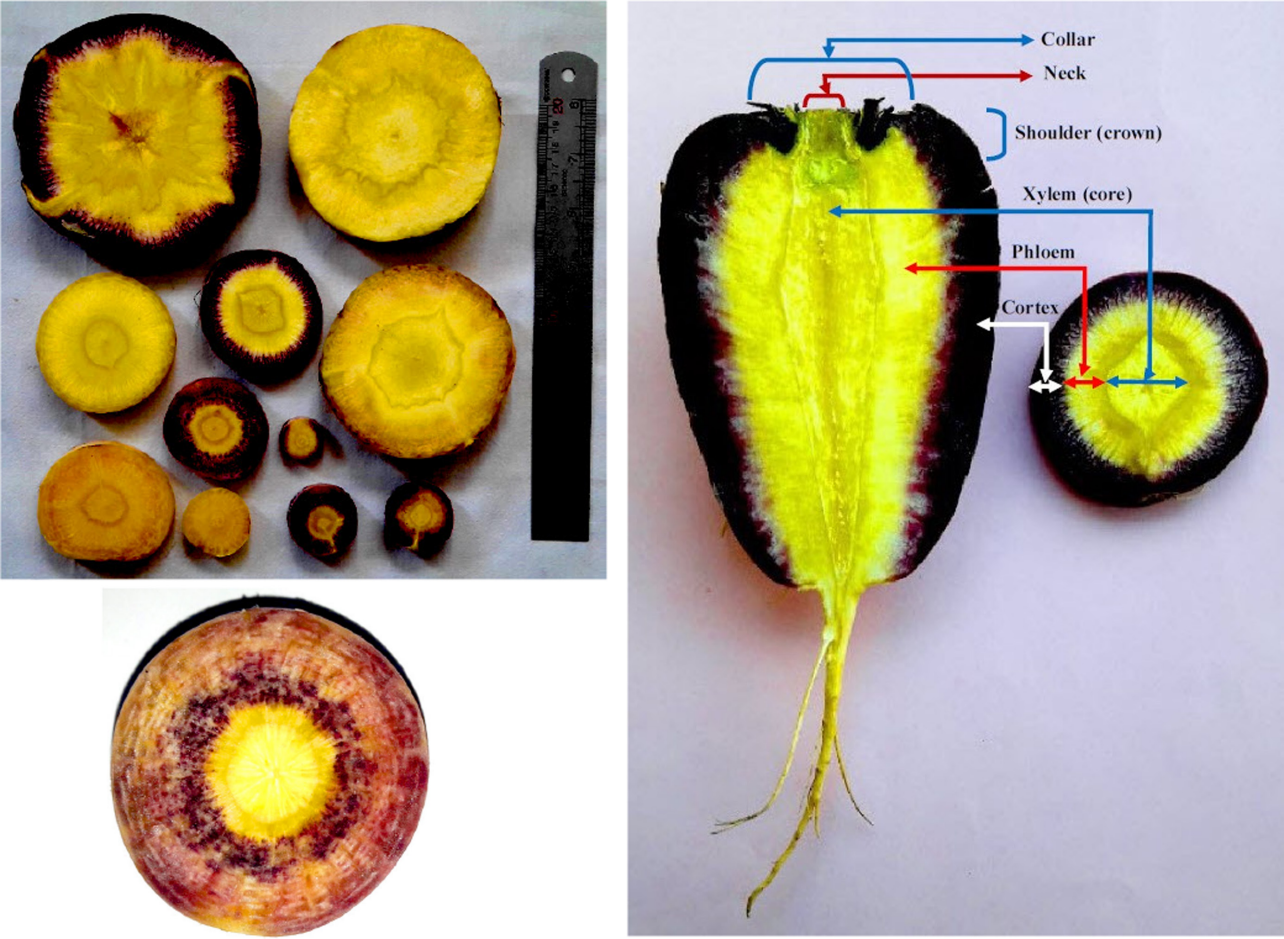
Inner sections of root of *P. sativa* accessions studied showing diversity in color.

Inner core (xylem) diameter at shoulder ranged from 6.02 to 64.57 mm, while outer core (phloem) thickness at shoulder varied from 3.32 to 20.32 mm. Total soluble solids varied between 7.30 and 16.50% with an average of 10.04%. Outer core (phloem) pigmentation/color was predominantly yellow (23 accessions), while outer core (cortex) pigmentation/color was predominantly dark purple (21 accessions). Inner core (xylem) pigmentation/color was cream yellow (11 accessions), light yellow (12), yellow (42), dark yellow (2), and yellow–light orange (2) (Figure 4). The different root color in carrot (*D. carota*) was conferred by the Y and Y2 loci in chromosomes 5 and 7, respectively, in which Y_Y2_, yyY2_, Y_y2y2, and yyy2y2 genotypes represent white, yellow, pale orange, and orange root color, respectively (Cavagnaro et al., 2011; Ellison et al., 2017). Moreover, the purple root color was due to the deposition of anthocyanin, which was regulated by the genes in the P1 and P3 regions of chromosome 3 (Bannoud et al., 2019, 2021). Color is an important quality parameter of fruits and vegetables evaluated by consumers as it highly affects their marketability (Pathare et al., 2013). In the present study, root color varied greatly among the genetic resources. Most of the accessions showed a differential range of color attributes between the outer and inner parts of the root. This information might be useful for selecting the desired colored carrots because an appropriate color increases consumer acceptance (Nisha et al., 2011).

Significant correlations were observed among some variables (Table 3). Foliage width (crown) showed significant and positive correlations with mature leaf length (*r* = 0.54), mature leaf width (*r* = 0.64), length of basal primary leaflet (*r* = 0.50), and petiole width (*r* = 0.51). Root length was significantly and positively correlated with root diameter at the middle point of the root (*r* = 0.55), root maximum transverse diameter (*r* = 0.53), taproot length (*r* = 0.35), neck diameter (*r* = 0.40), and collar diameter (*r* = 0.29). Root weight showed significant and positive correlations with foliage width (*r* = 0.52), mature leaf width (*r* = 0.54), length of basal primary leaflet (*r* = 0.35), petiole width (*r* = 0.43), petiole thickness (*r* = 0.52), root length (*r* = 0.66), and root diameter at the middle point of the root (*r* = 0.89). Total soluble solids were significantly and negatively correlated with root diameter at the middle point of the root (*r* = −0.39), root maximum transverse diameter (*r* = −0.42), taproot length (*r* = −0.32), root diameter at shoulder (*r* = −0.43), and root weight (*r* = −0.43).

**TABLE 3:** Simple correlations among the quantitative morphological variables utilized in the studied *P. sativa* accessions.

Character	V3	V6	V7	V15	V17	V18	V19	V30	V31	V32	V35	V36	V37	V38	V42	V51	V53	V54	V55	V67
V3	1																			
V6	0.54**	1																		
V7	0.64**	0.51**	1																	
V15	0.50**	0.58**	0.51**	1																
V17	0.02	0.65**	0.00	0.29*	1															
V18	0.51**	0.18	0.61**	0.20	−0.32**	1														
V19	0.57**	0.07	0.60**	0.37**	−0.44**	0.63**	1													
V30	0.35**	0.19	0.21	0.19	−0.08	0.14	0.33**	1												
V31	0.51**	0.09	0.51**	0.28*	−0.45**	0.43**	0.55**	0.46**	1											
V32	0.59**	0.26*	0.55**	0.35**	−0.29*	0.38**	0.53**	0.57**	0.93**	1										
V35	0.41**	0.16	0.35**	0.25*	−0.27*	0.38**	0.35**	0.27*	0.51**	0.49**	1									
V36	0.42**	0.28*	0.32**	0.37**	−0.10	0.34**	0.40**	0.56**	0.55**	0.59**	0.47**	1								
V37	0.42**	0.35**	0.39**	0.42**	0.04	0.24*	0.29*	0.51**	0.61**	0.73**	0.38**	0.76**	1							
V38	0.49**	0.23	0.40**	0.38**	−0.16	0.29*	0.42**	0.55**	0.82**	0.87**	0.46**	0.76**	0.89**	1						
V42	0.52**	0.23	0.54**	0.35**	−0.28*	0.43**	0.52**	0.66**	0.89**	0.92**	0.50**	0.67**	0.75**	0.86**	1					
V51	0.48**	0.26*	0.45**	0.34**	−0.14	0.35**	0.40**	0.61**	0.82**	0.86**	0.46**	0.75**	0.80**	0.90**	0.92**	1				
V53	0.40**	0.00	0.29*	0.24*	−0.33**	0.23	0.43**	0.39**	0.77**	0.75**	0.43**	0.49**	0.60**	0.81**	0.67**	0.64**	1			
V54	0.36**	0.06	0.29*	0.23	−0.28*	0.22	0.38**	0.30*	0.69**	0.67**	0.37**	0.50**	0.57**	0.73**	0.57**	0.56**	0.81**	1		
V55	0.29*	0.25*	0.32**	0.25*	0.06	0.19	0.14	0.08	0.36**	0.39**	0.20	0.39**	0.47**	0.52**	0.36**	0.38**	0.23	0.16	1	
V67	−0.23*	−0.22	−0.22	−0.07	0.03	−0.09	0.03	−0.18	−0.39**	−0.42**	−0.32**	−0.20	−0.29*	−0.35**	−0.43**	−0.36**	−0.30*	−0.24*	−0.26*	1

*Note*: For the explanation of the character symbols, see Table 1. *, **. Correlation is significant at *p* ≤ .05 and 0.01 levels, respectively.

**FIGURE 5:**
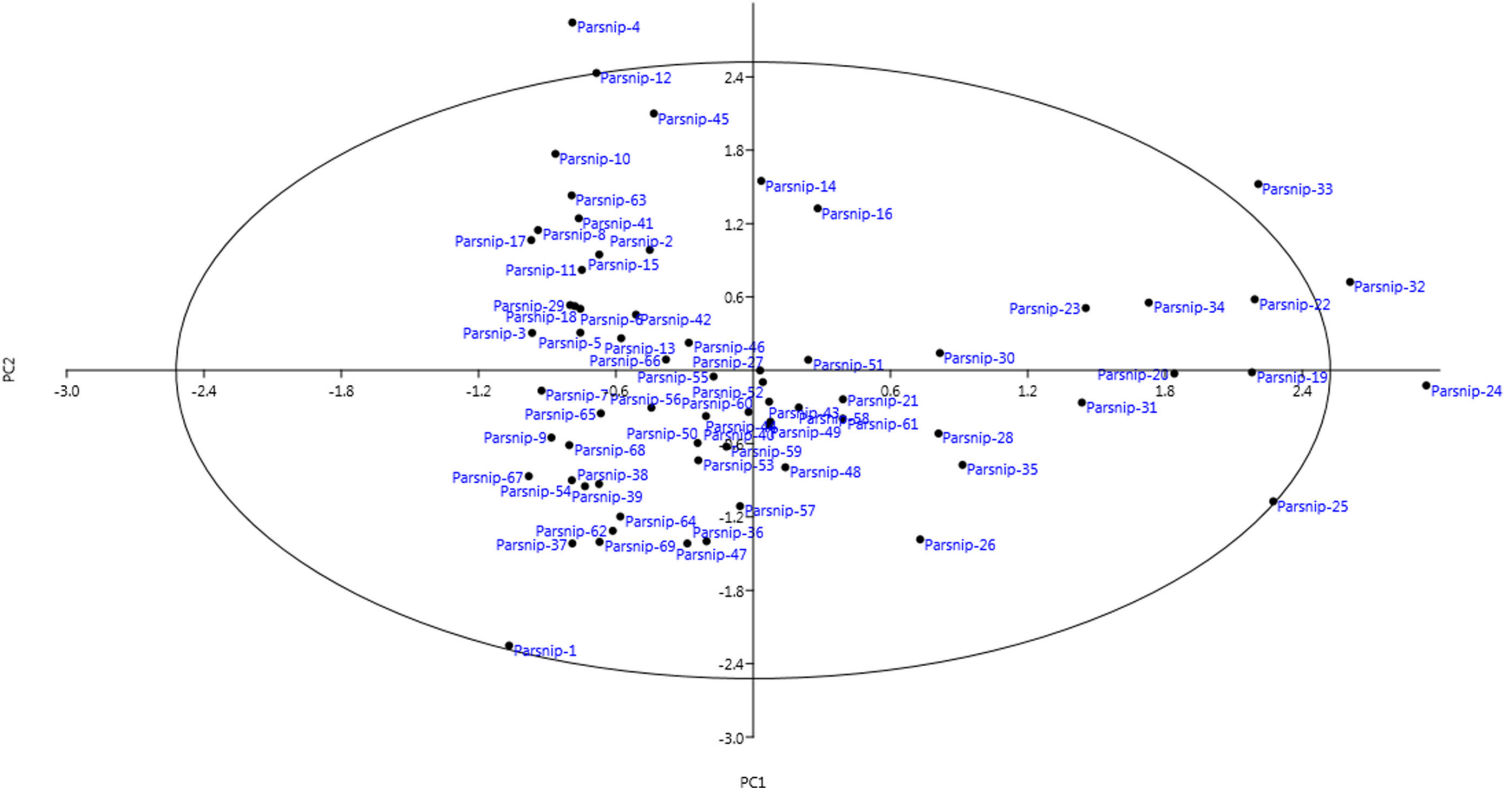
Scatter plot for the studied *P. sativa* accessions based on PC1/PC2.

The PCA showed 18 independent components that explained 81.98% of the total variance (Table 4). The PC1 showed positive correlations with number of mature leaves per plant, root length, root diameter at the middle point of the root, root maximum transverse diameter, neck diameter, collar diameter, root diameter at shoulder, root weight, inner core (xylem) diameter at shoulder, inner core (xylem) diameter at root maximum transverse diameter, root diameter of core (xylem) relative to total diameter, outer core (phloem) thickness at shoulder, and outer core (phloem) thickness at root maximum transverse diameter that explained 22.25% of the total variance. Root shape, root tapering, and root tip/end shape were loaded on PC2 and accounted for 7.45% of the total variance. The PC3 was correlated with mature leaf length, length of basal primary leaflet, number of segment tips on lower primary leaflet, and petiole length, accounting for 6.98% of the total variance. Based on the scatter plot generated using PC1 and PC2, the accessions were placed into four groups and most of them were placed in the center of plot (Figure 5). PCA has been previously used to investigate the phenotypic diversity of most plants (Khadivi et al., 2021; Khadivi, Mirheidari, & Moradi, 2022; Khadivi, Mirheidari, Saeidifar, & Moradi, 2022; Khadivi & Mirheidari, 2021; Moradi et al., 2022; Safdari & Khadivi, 2021).

**TABLE 4:** Eigenvalues of the principal component axes from the PCA of the morphological characters in the studied *P. sativa* accessions.

Character	Component																	
	1	2	3	4	5	6	7	8	9	10	11	12	13	14	15	16	17	18
V2	0.35	0.24	−0.01	0.01	**0.77**	−0.05	0.04	0.14	−0.14	−0.09	0.09	−0.05	−0.01	−0.02	0.10	0.03	−0.02	0.01
V3	0.45	0.06	0.52	−0.01	0.43	0.05	−0.04	0.10	−0.09	−0.02	0.21	0.16	0.01	0.04	0.05	−0.21	−0.17	−0.12
V4	−0.24	−0.20	0.26	0.00	−0.53	−0.14	0.49	0.10	0.03	−0.13	−0.21	0.01	−0.21	0.08	−0.06	0.04	−0.11	−0.13
V5	**0.59**	−0.08	−0.17	0.05	0.57	−0.03	−0.04	0.07	0.01	−0.06	0.00	−0.16	−0.20	−0.07	0.05	0.06	−0.02	0.00
V6	0.23	−0.08	**0.83**	−0.07	−0.07	0.02	0.14	0.19	0.07	0.02	−0.01	0.07	−0.07	−0.03	−0.10	0.07	−0.08	−0.16
V7	0.34	0.20	0.55	0.11	0.38	0.04	−0.07	0.37	−0.10	0.08	0.09	0.03	−0.13	0.04	−0.15	−0.15	0.08	0.03
V8	0.16	0.02	0.00	0.14	0.02	0.03	−0.13	**0.89**	0.07	0.07	−0.02	−0.02	0.13	−0.12	0.05	0.01	0.10	−0.05
V9	−0.33	−0.14	0.01	−0.22	−0.12	−0.01	0.08	0.03	−0.24	0.05	0.10	0.07	0.14	**0.69**	−0.17	−0.09	0.13	−0.02
V10	−0.28	−0.20	0.27	0.21	0.02	−0.23	0.20	−0.13	−0.05	−0.04	−0.17	0.07	−0.23	**0.63**	0.11	0.03	0.04	−0.06
V11	0.11	0.17	−0.07	0.10	0.04	0.07	−0.04	0.03	−0.14	0.02	0.17	0.08	**0.85**	−0.06	0.04	−0.05	0.04	0.08
V12	0.17	0.01	0.09	0.06	0.13	0.04	0.06	−0.13	0.07	0.07	**0.81**	−0.15	0.16	−0.06	0.00	0.09	−0.08	0.05
V13	0.12	0.27	−0.15	−0.09	0.28	−0.28	0.00	0.04	−0.02	0.04	**0.70**	0.16	0.04	0.07	−0.02	−0.18	0.07	−0.06
V14	0.44	0.25	0.40	0.00	0.09	0.12	−0.02	0.09	−0.09	−0.04	0.23	−0.01	0.02	0.07	0.41	−0.21	0.16	0.03
V15	0.31	−0.06	**0.71**	0.10	0.16	−0.27	−0.10	0.06	0.08	−0.03	−0.03	−0.01	0.23	0.06	0.06	−0.02	0.07	0.16
V16	0.09	0.09	**0.72**	0.00	−0.03	0.00	−0.29	−0.32	0.11	0.03	0.11	0.06	−0.02	0.18	0.11	0.13	0.00	0.13
V17	−0.16	−0.24	**0.64**	−0.12	−0.40	−0.15	0.31	−0.07	0.16	0.08	−0.24	0.02	−0.12	−0.01	0.01	0.02	−0.04	−0.08
V18	0.25	0.19	0.09	0.12	0.55	−0.02	−0.03	0.38	0.07	0.09	0.25	0.34	−0.16	−0.12	−0.15	0.08	0.04	−0.04
V19	0.33	0.12	0.12	0.27	**0.66**	0.00	−0.15	0.26	0.05	−0.03	0.20	0.07	0.22	−0.07	−0.03	−0.06	0.04	0.08
V20	−0.03	0.07	0.55	−0.22	−0.07	0.08	−0.14	0.05	0.00	0.23	−0.05	0.17	−0.25	−0.04	0.38	−0.32	0.13	−0.04
V21	−0.09	−0.13	0.16	−0.08	0.10	0.05	0.25	0.38	−0.09	0.17	0.38	0.28	−0.31	−0.09	0.22	−0.33	0.17	−0.10
V22	0.05	−0.06	0.08	−0.05	0.21	−0.09	−0.03	**0.86**	0.05	0.01	−0.07	−0.11	−0.07	0.16	0.00	0.10	0.01	0.04
V23	−0.05	0.01	−0.05	−0.01	−0.08	0.10	**0.85**	−0.15	−0.05	−0.22	0.11	0.03	−0.09	0.16	−0.01	0.07	−0.04	0.03
V24	0.04	0.01	−0.02	0.09	0.23	0.08	0.09	0.09	−0.37	−0.04	−0.01	0.02	−0.05	0.06	−0.15	−0.07	**0.72**	0.10
V25	0.02	0.19	−0.01	0.00	−0.24	−0.03	−0.05	0.15	**0.69**	−0.07	0.11	−0.06	−0.18	−0.07	−0.03	−0.08	−0.24	0.07
V26	0.25	0.05	0.05	0.01	−0.04	−0.13	0.07	−0.03	**0.88**	0.05	−0.04	0.01	−0.04	−0.10	0.00	0.01	0.01	0.08
V27	0.06	−0.02	0.11	0.04	0.11	0.21	−0.03	0.03	**0.87**	−0.09	0.00	0.00	0.03	0.01	0.00	0.10	−0.07	−0.07
V28	−0.07	0.01	−0.02	0.11	0.21	0.30	0.17	−0.08	0.01	−0.04	0.02	−0.13	−0.09	−0.02	−0.17	−0.02	**###**	0.12
V29	0.17	0.00	−0.02	0.06	0.02	−0.04	0.09	−0.03	0.05	−0.10	0.00	−0.01	0.06	−0.05	−0.03	0.03	−0.04	**0.84**
V30	**0.67**	−0.25	0.04	0.05	0.19	0.21	0.41	−0.01	−0.09	−0.06	0.13	−0.18	0.18	−0.06	0.08	0.05	−0.01	0.11
V31	**0.80**	0.36	−0.02	0.11	0.28	0.11	−0.19	0.03	−0.07	0.02	0.14	−0.04	−0.02	0.04	0.04	−0.03	0.07	0.00
V32	**0.87**	0.19	0.12	0.11	0.20	0.10	−0.15	0.06	−0.11	−0.01	0.14	0.00	−0.01	0.08	0.09	−0.04	0.03	−0.04
V33	−0.13	0.56	−0.10	−0.17	0.26	0.06	−0.08	−0.09	−0.05	−0.07	0.07	−0.06	−0.13	−0.19	−0.39	−0.07	0.07	0.02
V34	−0.35	−0.38	−0.09	−0.10	−0.07	−0.02	**0.72**	−0.07	0.11	−0.07	−0.05	−0.16	0.11	−0.05	−0.03	0.06	−0.02	0.16
V35	0.46	0.19	0.09	0.10	0.16	−0.03	−0.17	0.20	−0.01	−0.08	0.25	−0.04	−0.23	0.04	0.19	0.17	−0.19	0.25
V36	**0.79**	−0.02	0.10	−0.04	0.07	−0.07	0.03	0.08	0.17	0.05	0.10	0.08	0.14	−0.25	−0.17	0.12	−0.15	0.09
V37	**0.87**	0.01	0.22	−0.01	−0.11	−0.08	−0.10	−0.01	0.10	0.05	−0.06	0.10	0.04	−0.01	0.01	0.07	−0.01	0.07
V38	**0.93**	0.20	0.10	0.08	0.03	−0.02	−0.09	−0.01	0.06	0.06	−0.02	0.05	0.06	−0.07	0.11	0.00	−0.04	0.04
V39	0.00	0.29	0.14	0.19	0.43	0.30	−0.12	−0.07	0.02	0.24	0.17	−0.28	0.10	−0.01	−0.16	0.10	−0.09	−0.05
V40	0.19	−0.02	−0.09	−0.24	−0.10	**0.80**	0.18	0.05	−0.04	0.00	0.00	−0.11	0.00	0.03	0.11	0.03	−0.13	−0.02
V41	0.14	0.05	−0.01	**0.87**	−0.03	−0.20	0.10	−0.01	0.02	0.13	0.09	−0.02	0.02	−0.07	−0.01	−0.03	−0.04	−0.01
V42	**0.90**	0.15	0.08	0.08	0.20	0.10	−0.02	0.11	0.03	0.00	0.09	−0.01	0.02	0.11	0.01	−0.05	0.11	0.06
V43	0.33	−0.06	−0.02	0.12	−0.25	0.13	0.11	0.02	0.06	−0.23	0.22	−0.03	0.04	0.08	−0.03	0.49	−0.09	0.22
V44	0.27	0.06	0.05	−0.03	0.00	−0.02	−0.05	−0.28	−0.03	−0.08	0.08	0.05	0.19	0.22	0.06	**−0.61**	0.00	−0.03
V45	0.27	**0.69**	−0.02	0.10	0.10	0.09	−0.17	0.05	0.09	−0.04	0.27	0.13	0.09	−0.05	−0.03	0.20	0.01	−0.13
V46	−0.38	**−0.81**	−0.01	0.02	−0.11	−0.01	0.07	0.00	−0.11	−0.02	0.04	−0.04	−0.18	0.01	−0.14	0.09	−0.06	−0.03
V47	−0.30	**###**	−0.01	0.00	−0.07	0.12	0.00	0.01	−0.06	−0.02	0.00	0.04	−0.03	−0.02	−0.07	0.11	0.09	−0.10
V48	0.19	0.04	−0.07	**0.83**	0.14	−0.38	−0.01	0.09	0.08	0.01	0.05	−0.02	−0.01	−0.02	−0.03	0.05	0.04	0.10
V49	0.03	−0.02	0.03	**0.83**	0.03	0.12	−0.11	0.06	−0.14	0.01	−0.12	0.07	0.20	0.04	0.06	0.01	−0.02	−0.03
V50	**0.92**	0.03	0.05	0.06	0.06	−0.05	0.03	0.05	0.22	0.09	0.02	0.02	−0.02	−0.06	−0.07	−0.06	0.10	0.07
V51	**0.94**	0.13	0.08	0.05	0.06	0.02	0.04	0.04	0.13	0.06	0.03	−0.02	−0.02	−0.03	−0.06	−0.08	0.08	0.03
V52	**0.62**	−0.05	−0.09	0.09	−0.07	0.04	0.45	0.11	−0.06	−0.02	0.04	0.03	0.05	0.01	−0.44	0.02	0.10	0.15
V53	**0.69**	0.29	−0.05	0.03	0.16	0.08	−0.19	−0.03	−0.02	0.01	0.03	−0.01	0.13	−0.13	0.47	−0.08	−0.06	−0.01
V54	**0.58**	0.46	0.07	−0.10	0.20	0.01	−0.20	−0.14	0.06	−0.04	0.05	−0.09	0.05	−0.24	0.32	0.12	−0.03	−0.07
V55	0.43	0.17	0.22	0.19	−0.14	0.00	−0.12	0.15	−0.14	0.35	−0.17	0.27	−0.03	0.11	−0.15	0.06	−0.18	0.07
V56	0.19	0.04	0.01	0.20	0.00	0.01	0.07	0.11	−0.04	0.31	−0.04	0.36	0.08	−0.06	0.42	−0.02	0.20	0.44
V57	0.01	0.03	0.13	0.08	0.00	−0.10	−0.06	−0.12	−0.02	−0.02	−0.02	**0.82**	0.08	0.03	0.03	−0.01	0.09	−0.01
V58	0.04	−0.08	−0.04	**0.80**	0.11	−0.36	−0.03	−0.02	0.12	−0.05	0.01	0.10	−0.12	−0.03	−0.01	0.00	0.01	0.10
V59	−0.08	−0.12	−0.11	−0.14	0.25	**0.71**	−0.04	−0.06	0.06	−0.17	−0.20	−0.02	−0.03	−0.14	−0.14	−0.07	0.16	0.02
V60	0.05	0.08	0.00	−0.27	−0.04	**0.85**	−0.05	−0.04	0.04	−0.02	0.04	−0.01	0.08	0.03	0.04	0.03	−0.11	−0.02
V61	0.16	0.15	−0.25	0.26	0.03	0.12	−0.11	−0.16	0.09	−0.18	−0.01	−0.32	−0.27	0.17	−0.23	0.34	0.20	0.24
V62	0.03	−0.05	0.13	−0.30	0.32	−0.13	0.15	−0.16	0.02	0.22	−0.05	0.12	0.16	0.14	0.09	0.55	−0.02	−0.26
V63	0.50	0.27	−0.04	0.19	0.20	−0.16	−0.13	−0.12	−0.01	−0.29	0.01	−0.01	0.13	−0.29	0.05	0.14	−0.10	0.03
V64	0.06	0.09	0.04	−0.05	0.02	−0.04	−0.05	0.16	−0.14	**0.69**	0.26	−0.31	0.09	−0.04	0.09	0.16	0.12	−0.18
V65	0.03	−0.10	0.04	0.02	0.04	0.05	−0.10	−0.06	0.03	**0.84**	−0.04	−0.03	0.04	0.03	−0.01	−0.09	−0.03	−0.07
V66	0.08	0.05	0.00	0.13	−0.07	−0.29	−0.16	0.09	−0.02	**0.72**	0.03	0.30	−0.13	0.06	0.03	0.06	−0.03	0.12
V67	−0.39	−0.22	−0.06	0.11	0.05	−0.11	0.01	−0.15	−0.01	−0.17	0.04	0.21	0.19	−0.46	−0.14	−0.01	0.16	0.13
Total	14.68	4.92	4.61	4.33	3.36	2.81	2.48	2.16	2.12	1.96	1.75	1.52	1.43	1.33	1.27	1.17	1.15	1.08
% of variance	22.25	7.45	6.98	6.56	5.09	4.25	3.76	3.27	3.21	2.97	2.65	2.30	2.16	2.02	1.92	1.78	1.74	1.63
Cumulative %	22.25	29.70	36.68	43.24	48.32	52.58	56.34	59.60	62.82	65.79	68.44	70.74	72.90	74.92	76.84	78.62	80.35	81.98

*Note*: Bold values indicate the characteristics most influencing PCs. For the explanation of the character symbols, see Table 1.

**FIGURE 6:**
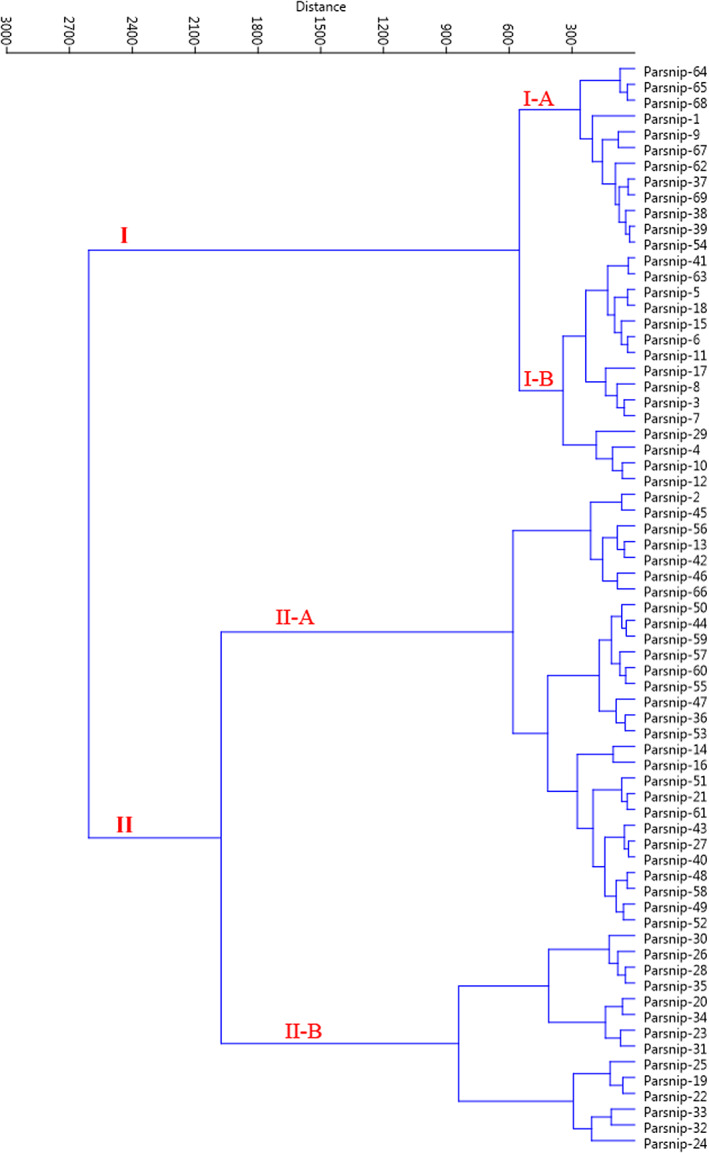
Ward cluster analysis of the studied *P. sativa* accessions based on morphological traits using Euclidean distances.

In the cluster analysis based on Ward's method, the accessions were divided into two main clusters according to morphological traits (Figure 6). The first cluster (I) consisted of 27 accessions, forming two sub‐clusters. Sub‐cluster I‐A consisted of 12 accessions and sub‐cluster I‐B contained 15 accessions. The rest of the accessions were classified into the second cluster (I), forming two sub‐clusters. Sub‐cluster II‐A included 28 accessions, while 14 accessions formed sub‐cluster II‐B. The discrepancy between the cluster and scatter dendrograms can be explained by the variability considered for the analysis. Cluster analysis was based on all the morphological data and took into account the whole variability, while the scatter plot was created using the cumulative variance of PC1 and PC2 and was relatively low (29.70%).

Forgotten plant species can play a significant role in providing food security and improving the quality level of nutrition which is a herald of food security. By removing these types of plants from the human food basket, its consequences will be determined according to the inevitable effects of climate change on food production (Koocheki et al., 2018). With the expansion of carrot cultivation, the attention to parsnip decreased and the cultivation of this medicinal plant is low. This is despite the fact that parsnip is part of the medicinal native plant and valuable in most farms in tropical cities. Compared with carrots, parsnip plants are more adaptable to different environmental conditions.

Based on ideal values of the important and commercial characters of parsnip, such as root length, root weight, inner core (xylem) pigmentation/color, root shape, flesh color intensity, flesh palatability, and total soluble solids, 14 accessions, including Parsnip‐3, Parsnip‐9, Parsnip‐24, Parsnip‐32, Parsnip‐32, Parsnip‐48, Parsnip‐51, Parsnip‐52, Parsnip‐58, Parsnip‐60, Parsnip‐62, Parsnip‐65, Parsnip‐67, and Parsnip‐69, were promising and are recommended for cultivation. These accessions could be considered for future breeding programs as yield is one of the parameters that is considered in commercial breeding programs (Tabor et al., 2016). However, analysis of seasonal and yearly variations might be useful for gathering information for stable production. The wide variability in both qualitative and quantitative traits found in this study might be useful for identifying genotypes and might be required for the genetic improvement of crops (Hooks et al., 2021; Luitel et al., 2018). It has been reported that differential expression of a number of genes located in chromosomes 1, 2, and 7 is responsible for the differences in root characteristics of carrots (Macko‐Podgorni et al., 2020; Turner et al., 2018). The selected accessions with higher weight and uniform size can be used for future breeding programs. Overall, the results of the present study might be useful for selecting candidate genotypes with better growth performance and higher yield. To the best of our knowledge, this is the first report showing morphological variability in parsnip genetic resources of diverse origins.

## 4 CONCLUSIONS

The best method of propagation of parsnips is mainly through seed. Parsnip seeds have a high healing power and do not require any treatment for germination, so it is recommended that the seeds of this plant are collected in the middle of summer and planted directly in the field. Due to unique features of this plant, such as adaptability, resistant to drought, heat, and salinity of water and soil, parsnip as an agricultural and medicinal plant is recommended for cultivation in fields with salty desert soils. In addition to numerous uses and applications for food and medicinal value of the roots of this native plant, the ringed leaves of parsnip can also be fed to livestock as fresh winter fodder (with high nutritional value). Also, for each hectare of fields cultivated with parsnip, average of 2 to 3 tons of seeds per hectare is harvested. The accessions studied here showed high phenotypic diversity and some of them can be selected and cultivated. Therefore, the promotion and development of parsnip plant cultivation, as a native plant adaptable to ecological conditions, is recommended. Based on ideal values of the important and commercial characters of parsnip, such as root length, root weight, inner core (xylem) pigmentation/color, root shape, flesh color intensity, flesh palatability, and total soluble solids, 14 genotypes, including Parsnip‐3, Parsnip‐9, Parsnip‐24, Parsnip‐32, Parsnip‐32, Parsnip‐48, Parsnip‐51, Parsnip‐52, Parsnip‐58, Parsnip‐60, Parsnip‐62, Parsnip‐65, Parsnip‐67, and Parsnip‐69, were promising and are recommended for cultivation.

## AUTHOR CONTRIBUTIONS

**Ali Khadivi:** Formal analysis (lead); investigation (equal); methodology (lead); software (lead); validation (lead); writing – original draft (lead); writing – review and editing (lead). **Farhad Mirheidari:** Investigation (equal). **Younes Moradi:** Investigation (equal).

## CONFLICT OF INTEREST STATEMENT

The authors declare no conflict of interest.

## Data Availability

The data that support the findings of this study are available from the corresponding author upon reasonable request.
